# Collaboration among emergency first responders at major incidents – an explorative focus group study

**DOI:** 10.1186/s13049-026-01606-4

**Published:** 2026-04-02

**Authors:** Andreas Rantala, Anna Conradsson, Jonathan Adamsson, Lena Forsell, Jonas Wihlborg

**Affiliations:** 1 Department of Health Sciences, Faculty of Medicine, Lund University, 221 00 Lund, Sweden; 2 Ambulance Service Department, Office of Medical Services, Region Skåne, Helsingborg, Sweden; 3 Centre of Interprofessional Collaboration Within Emergency Care (CICE), Linnaeus University, Växjö, Sweden; 4 Ambulance Service Department, Region Jönköpings Län, Sweden; 5 Department of Prehospital Emergency Care, Sahlgrenska University Hospital, Gothenburg, Sweden; 6 School of Health and Welfare, Dalarna University, Falun, Sweden

**Keywords:** Ambulance, Ambulance nurse, Collaboration, Content analysis, Emergency first responders, EMS, Fire brigade, Police

## Abstract

**Background:**

In the event of a major incident, collaboration between the ambulance service, fire brigade and police is common. Effective collaboration requires teamwork, with communication between the three organisations being crucial. Leadership functions should be integrated in a way that leverages organisation-specific capabilities, enabling interprofessional collaboration where diverse professional roles complement each other. The aim of this study is to explore the experiences of collaboration among emergency first responders during major incidents.

**Methods:**

Data were collected through four focus group interviews involving ambulance nurses, firefighters and police officers, each with a minimum of one year of operational experience. The data were analysed using content analysis inspired by Krueger and Casey.

**Results:**

Three categories emerged from the analysis; *Coordinated communication as a foundation for collaboration*, *Structured interprofessional collaboration through leadership and shared understanding* and *Relational conditions for sustained interprofessional collaboration*. Each category comprises two subcategories that reflect the participants’ experiences.

**Conclusion:**

The findings highlight that collaboration among emergency first responders during major incidents is shaped by coordinated communication, structured leadership and mutual understanding of organisational roles. Communication challenges, leadership demands and safety considerations in complex incidents were described as influencing collaboration. Joint training and interprofessional interaction may support preparedness and strengthen collaboration across emergency service organisations.

**Supplementary Information:**

The online version contains supplementary material available at 10.1186/s13049-026-01606-4.

## Introduction

In an increasingly interconnected world, societal infrastructures, supply-chains, and digital networks are tightly interdependent. Consequently, disturbances in one sector more readily cascade into others, a phenomenon often described as cascading or systemic disasters, contributing to both a higher frequency of major incidents and a marked increase in their complexity [1]. As a result, the frequency and complexity of major incidents have escalated, for example, natural disasters are forecast to increase by as much as 40 percent, presenting significant challenges for emergency response systems globally [2]. Major incidents encompass a broad spectrum of events, including serious or large-scale accidents, natural disasters and deliberate acts of violence that cause harm and suffering to individuals and communities [2–4]. They can be described as an event or situation with a range of serious consequences, requiring special arrangements to be implemented by one or more emergency responder agencies [5]. These incidents pose not only immediate physical threats but also psychological repercussions for those involved [6], including bystanders [7].

Effective management of major incidents is therefore critical, necessitating a coordinated response from the emergency services [8], namely the ambulance service, police and fire brigade. In Sweden, these emergency first responder organisations are collectively referred to as blue light authorities.

Collaboration between emergency first responders is essential for mitigating the impact of major incidents [9]. However, in Sweden, as in many other jurisdictions, these organisations operate under separate legal frameworks: the municipalities are responsible for the fire brigade, the ambulance service falls under the jurisdiction of the 21 regions, while the police is a state agency. These differences in legislation and operational protocols can lead to ambiguities regarding roles and responsibilities during emergencies, potentially hindering timely and effective responses [10]. Such lack of clarity may undermine the effectiveness of the response and increase the risks faced by both victims and responders [11, 12]. In addition, a persistent gap between the ideal of collaboration and what is achievable in practice has been demonstrated, where uncertainty, power asymmetries and organisational boundaries can limit joint action at the scene [13, 14]. Research indicates that the initial response to a major incident, particularly within the first hour, is crucial for saving lives and minimising harm [15]. This period, often referred to as the "golden hour", is characterised by heightened urgency, where swift and coordinated action can significantly improve victim outcomes. Delays or miscommunication during this critical phase may result in detrimental consequences, including increased morbidity and mortality rates [16]. Fostering a culture of collaboration is therefore essential to ensure that first responders operate as an integrated team, effectively leveraging their collective resources and expertise [14].

Interprofessional collaboration has also been emphasised in a UK study on multi-agency response (i.e., ambulance service, fire brigade and police). The study explored the relationship between shared identity and improved group functioning, highlighting the importance of a shared frame of reference in developing a collective identity that can enhance training and management for first responders [17]. Studies from Nordic prehospital settings indicate that designated on-scene medical leadership and senior ambulance management roles can strengthen coordination, trust and medical decision-making in complex incidents [11, 18]. At the same time, variability in disaster preparedness and training among prehospital professionals highlights ongoing challenges to effective collaboration under pressure [19], while research on police led high-threat events underscores the operational necessity of interoperable work across emergency services [20]

It has been demonstrated that successful responses depend on clear leadership structures, shared situational awareness and joint preparedness activities[21–23]. In Sweden, regional disparities in prehospital organization and limited frequency of multi-agency exercises undermine collaborative capacity, emphasizing the need for standardized national guidelines and recurring joint drills [21]. While training programmes provide technical frameworks such as triage and reporting, they often neglect the interpersonal and adaptive skills essential for collaboration under stress, including communication, ethical decision-making and resilience [24]. Interprofessional exercises foster trust and confidence among responders, yet structured feedback and non-technical skill development remain insufficient [25]. Evidence from UK terrorist incidents demonstrates that teamwork and innovative collaborative practices, such as co-locating blood bank staff in emergency departments and sharing resources across the trauma network, significantly enhance system performance, whereas fragmented communication and lack of psychosocial support hinder it [22]. At a strategic level, robust hospital plans, tested through realistic simulations, ensure coordination between emergency services and healthcare systems. Without such collaborative planning and recovery strategies, even well-resourced systems risk failure during crises [23].

Communication plays a vital role in this collaborative context. Studies have shown that effective communication is integral to the success of interprofessional teams during emergencies [11, 26]. This emphasises the importance of robust communication networks, boundary-spanning roles and shared situational understanding to for enabling adaptive coordination in dynamic and high-risk contexts [27]. Clear and timely information exchange not only facilitates coordinated action but also enhances situational awareness among responders, enabling informed decision-making in high-pressure environments [28, 29]. Conversely, poor communication can increase confusion and delay responses [30], ultimately compromising patient safety.

Given the prevalence of major incidents, it is essential to examine the nature of collaboration among emergency first responders. The significance of timely and coordinated action, coupled with the critical role of communication, underscores the need for further research into the dynamics of collaboration among these organisations. Accordingly, this study aims to explore the experiences of collaboration among emergency first responders during major incidents.

## Methods

### Design

This study employed a qualitative methodology with an inductive approach. Data were generated through semi-structured focus group interviews and analysed using qualitative content analysis [31]. Focus groups were chosen as the data collection method as the study aimed to explore emergency first responders’ experiences of interagency collaboration during major incidents. Such collaboration involves complex organisational and relational processes that are often shaped through interaction between professional groups. Focus group discussions enable participants to reflect on and respond to each other’s experiences, thereby facilitating the exploration of shared practices, differing perspectives and collective understandings of collaboration [32]. Participants were therefore brought together in groups representing different emergency services to stimulate discussion and reflection on interagency collaboration from multiple professional perspectives. The interaction within the groups allowed participants to elaborate on experiences, clarify viewpoints and build on each other’s accounts, thereby enriching the data generated. The discussions were guided by open thematic areas**,** while allowing participants’ reflections and interactions to shape the direction of the conversation in line with the inductive approach [33].

The methodological reporting adheres to the Consolidated Criteria for Reporting Qualitative Research (COREQ) guidelines [34].

### Selection and participants

Purposive sampling was used to ensure variation in participants’ age, sex and professional experience [35]. Inclusion criteria required participants to be specialist ambulance nurses, firefighters, or police officers, each with a minimum of one year of operational service. Furthermore, participants were required to have direct operational experience of interagency collaboration during a major incident, defined as an event or situation with a range of serious consequences requiring special organisational arrangements by one or more emergency responder agencies [5]. The incidents described by participants included large-scale traffic collisions, fires, incidents involving multiple casualties and situations with ongoing lethal violence, all requiring a coordinated multi-agency response. Recruitment was conducted across two regions in southern Sweden. The sample size was guided by the concept of information power as described by Malterud et al. [36], whereby the more relevant information the sample holds, the fewer participants are required. A total of 23 participants were included and a summary of participant demographics is presented in Table 1.

**Table 1:** Demographic data of the participants in the focus group study (N = 23)

Variables	Total number of participants in Focus Groups (N = 23) n (%)	Focus Group 1 (n = 5) n (%)	Focus Group 2 (n = 4) n (%)	Focus Group 3 (n = 7) n (%)	Focus Group 4 (n = 7) n (%)
Sex *Male* *Female*	15 (65) 8 (35)	2 (40) 3 (60)	2 (50) 2 (50)	5 (71) 2 (29)	6 (86) 1 (14)
Age median (range)	37 (23–56)	35 (29–54)	33 (28–50)	39 (23–53)	36 (24–53)
Profession					
*Ambulance Nurse*	10 (44)	2 (40)	2 (50)	3 (43)	3 (42)
*Fire Fighter*	6 (26)	1 (20)	0 (0)	3 (43)	2 (29)
*Police Officer*	7 (30)	2 (40)	2 (50)	1(14)	2 (29)
Work experience in years, median (range)	7 (2–33)	9 (5–18)	6 (2–27)	12 (3–25)	5 (2–33)

### Data collection

Operations managers from the ambulance service, fire brigades and police in two regions of southern Sweden were contacted to obtain approval for participant recruitment. Once approval was granted, station managers or equivalent personnel were approached to jointly review the inclusion criteria. Subsequently, letters of interest were distributed to employees who met the criteria. Individuals who wished to participate contacted the authors [AC, JA] for further information.

A pilot interview was conducted with one participant to ensure that the interview questions aligned with the aim of the study [35]. This interview was not included in the final analysis, although no amendments were made based on its outcome. Four focus group interviews were conducted, each comprising four to seven participants. The semi-structured interviews were carried out by [AC and JA] using digital means via LU-Zoom. One of the interviewers had previous operational experience within the EMS setting. However, prior to data collection, the team discussed and reflected on this pre-understanding and potential sources of bias to ensure awareness and mitigate its potential influence. The interviewers had only limited experience in conducting focus group discussions but were under the continuous supervision of the first author [AR], who has extensive expertise in qualitative research. During the interviews, one author facilitated the interview, while the other served as an observer, taking notes on interactions and emergent themes to provide a summary at the end of each session. The interview commenced with the main opening question: “When we say collaboration between the ambulance service, police and fire brigade during major incidents, what comes to mind?” The discussion then proceeded through several open thematic areas concerning participants’ experiences of interagency collaboration during major incidents, aspects perceived as important for well-functioning collaboration and reflections on situations where collaboration had worked well or less well. For example, participants were invited to reflect on questions such as: “What experiences have you had of collaboration in these situations?” and “What do you perceive as important for well-functioning collaboration during major incidents?” Open-ended prompts (e.g., “Can you give an example?”, “In what context might this occur?” and “Could you elaborate?”) were used to clarify and deepen reflections. Particular emphasis was placed on encouraging participants to engage with and reflect on each other’s experiences, allowing perspectives to emerge inductively through the group dialogue [31]. The focus group interviews were only audio was recorded using external equipment and the recordings were transcribed verbatim. All participants were pseudonymised to ensure confidentiality.

### Data analysis

The analysis was inspired by Krueger and Casey’s [31] model of content analysis for focus groups and followed a structured approach to ensure rigour and trustworthiness [37]. Initially, all authors read the interview transcripts multiple times. Meaning units relevant to the study aim were identified, condensed and coded. The codes were then grouped into subcategories, which were further refined and summarised, forming the basis for broader categories. These categories provided answers that addressed the research aim and served as a guiding framework throughout the analysis to ensure the relevance and coherence of the findings [31]. An example of the analysis process is presented in Table 2.

**Table 2:** Example of the analysis process

Category	Subcategory	Code	Meaning Unit
Coordinated commmunication as a foundation for collaboration	Communication is central to the operation	Communication ensures structure Waiting for instructions promotes order Scene overview enables task delegation Dialogue determines who does what	Without clear communication, and if it’s a major incident scene, it’s important to wait your turn until those who have formed a clearer picture and have been on site longer can delegate tasks. That’s how I see it, from my perspective
Structured interprofessional collaboration through leadership and shared understanding	Role understanding across professional boundaries	Mutual trust is crucial Understanding professional differences Respect for others' ways of working Acceptance of different priorities	Trust is essential, as well as understanding that each profession has its own ways of working and priorities

### Ethical considerations

This study adhered to the ethical code of conduct and followed the guidelines issued by the Swedish Research Council. Key ethical principles, including consent, confidentiality, utility and transparency, were considered in accordance with the Declaration of Helsinki [38]. All participants received written information about the study and provided informed written consent prior to participation. The study was reviewed by the Regional Ethical Review Authority in Sweden, which issued an advisory opinion (Dnr 2021–03981).

## Results

Based on the analysis of the four focus group sessions, three overarching interrelated categories were identified: Coordinated communication as a foundation for collaboration, Structured interprofessional collaboration through leadership and shared understanding and Relational conditions for sustained interprofessional collaboration. Each category comprises two subcategories, which are presented in detail below and illustrated by quotations from participants (indicated by participant number in brackets, where F stands for Firefighter, P for Police officer and A for Ambulance nurse, followed by the focus group number). Categories as well as subcategories are numbered to improve the overall structure for readers. A summary of these categories and subcategories is provided in Table 3.

**Table 3:** Summary of categories and subcategories

Categories	Subcategories
1.0 Coordinated communication as a foundation for collaboration	• 1.1 Communication is central to the operation • 1.2 The impact of response time on collaboration
2.0 Structured interprofessional collaboration through leadership and shared understanding	• 2.1 Operational leadership for successful collaboration • 2.2 Role understanding across professional boundaries
3.0 Relational conditions for sustained interprofessional collaboration	• 3.1 Co-location promotes collaboration • 3.2 Post-incident reflection and shared learning

## Coordinated communication as a foundation for collaboration

This category highlights how coordinated communication, both prior to and following arrival at the scene, shapes first responders’ ability to collaborate effectively at major incidents. It underscores the importance of a shared language, reliable communication systems and timely information exchange, both prior to and following arrival at the site of a major incident. Delayed arrival without adequate information may lead to role confusion or reduced operational effectiveness. This category includes two subcategories: *Communication is Central to the Operation* and *The Impact of Response Time on Collaboration.*

### Communication is central to the operation

Communication was described as a key element enabling the operation to function effectively, both within individual organisations and across professional boundaries. Upon arrival at the scene, communication typically occurs via radio. Facts and instructions are transmitted to guide incoming units on where and how to deploy their efforts. Initial brief and concise radio communication was considered crucial for establishing a shared situational awareness among all incoming units. To support mutual understanding, the use of common terminology was emphasised. This included structured reporting tools applied in prehospital medical management, such as METHANE (a checklist/memory aid for reporting when arriving at the scene), verification reports, closed-loop communication to confirm message receipt and SBAR (a structured model for information transfer).

Participants reported challenges with cross-organisational radio channels, those used jointly by emergency services. Problems arose when the wrong channel was selected or when information was transmitted across multiple channels, while the intended recipient had access to only one radio device and thus only one channel at a time. In addition to pre-arrival communication, participants emphasised the importance of clear and coordinated communication when new staff arrive at the scene. They noted that each emergency service organisation has distinct information needs, which must be acknowledged to ensure efficient task allocation and situational awareness.

*Clear communication and finding the right person to talk to… If everyone just jumps out of their ambulances and rushes in to take whoever they think is the highest priority, then it doesn't work out well. There needs to be clear communication and if it’s a large incident scene you have to wait until those who have been there longer can delegate tasks.* (A10)

### The impact of response time on collaboration

The findings indicated that collaboration begins even before arrival at the scene, as information broadcast over the radio provides responders with an initial understanding of the situation. A longer response time was described as both a challenge and an opportunity. Participants expressed frustration about arriving late, being unable to begin work immediately and knowing that injured individuals were in need of assistance. Frustration also arose when tasks had already been completed by another organisation, leaving those who arrived later feeling redundant. However, a longer response time was also seen as beneficial in some cases, as it allowed personnel to feel more prepared upon arrival, thereby facilitating smoother collaboration. Participants emphasised the importance of receiving as much information as possible before reaching the scene.

*Even though it negatively affects the third party that we have a longer response time, it can still be beneficial to have a little more preparation.* (A1)

## Structured interprofessional collaboration through leadership and shared role understanding

This category illustrates how interprofessional collaboration is structured through both formal leadership arrangements and a shared understanding of organisational roles. Together, these dimensions shape decision-making authority, task allocation and collaborative safety considerations during major incidents. The category includes two subcategories*: Operational Leadership for Successful Collaboration* and * Role understanding across professional boundaries.*

### Operational leadership for successful collaboration

Participants emphasised that leadership is essential for effective collaboration during major incidents, as they require a different approach compared to routine operations. A structured command hierarchy, with clearly designated leaders, was considered critical. Having clinically experienced and task-trained individuals within each emergency service organisation was viewed as a positive factor that supports collaboration. Participants noted that, unlike the fire brigade, the ambulance service and police often lack a pre-established command structure, which can hinder operational leadership. To strengthen leadership capabilities, participants stressed the importance of applying consistent command structures even in minor incidents, i.e., events that can be managed within the ordinary routines and resources. This allows those in leadership roles to develop their skills over time. Collaboration at command level was reported to be effective when the leadership role was assumed by someone willing to lead actively and engage with other organisations throughout the operation. Regular in-person meetings between command personnel from all involved services were considered a key factor for success.

*However, the best communication and collaboration occur on-site when we all walk up together, everyone from the different organisations, look each other in the eye and talk. That’s when things work*. (A1)

The availability of personnel on-site was also seen to influence the command structure. The ambulance service, in particular, reported challenges when resources were limited. For example, standard procedure dictates that the first ambulance on the scene should establish a command function rather than engage in clinical work. However, a shortage of personnel may require the individual responsible for the command function to participate in patient care, although this was not seen as compromising patient safety. Participants also noted that prehospital medical management becomes more complex during incidents involving ongoing lethal violence, due to uncertainty around safety and decision-making responsibilities. In such contexts, command and collaboration become more challenging, reinforcing the need for structured radio communication and flexible role allocation across organisations.

*Then maybe ambulance personnel don't need to drive, instead we can take on roles that do not require extensive training in healthcare, allowing ambulance personnel to focus on what they do best. I thought this was a very good example of when roles are mixed and filled with the most effective functions.* (P5)

### Role understanding across professional boundaries

This subcategory describes how relational role understanding across professional boundaries enables mutual respect, flexible task allocation and effective collaboration during major incidents. Understanding the capabilities, responsibilities and legal frameworks of each organisation was described as essential for coordinating efforts and prioritising tasks. These organisation-specific competencies guided task prioritisation during operations. Supporting and assisting each other was considered a natural part of the work, particularly when tasks were coordinated in advance. Participants described a shared mindset focused on achieving a common goal, grounded in humility and mutual role comprehension.

*What happens at the scene is really just about coordinating ourselves. If it’s something for healthcare or the fire brigade, we try to support with what we can—unless we have something that clearly falls within our area of responsibility before it officially becomes our task, so to speak. It’s simply about coordinating and helping each other out*. (P1)

*Yes, I feel the same. I think it’s really about collaboration—working together as well as possible and forming a picture based on everyone’s different professions and what needs to be prioritised*. (A1)

*I completely agree. It’s about asking: what’s the most important thing right now? Who can best provide that support? And how can we assist that person or organisation in the best possible way? It’s also important that we actually talk to each other and sort things out. Everyone does their best to make things run as smoothly as possible for what’s needed right there and then.* (F1)

Humility, mutual respect, responsiveness and trust were considered essential for fostering an interprofessional team spirit and a supportive working environment. Non-prestigious collaboration, where organisations work together without hierarchy or competition, was seen as beneficial for achieving shared goals. Establishing a common objective early in the operation helped focus efforts and ensured that each organisation understood its role in the broader context. This shared situational awareness enabled task prioritisation based on available resources, with all services ultimately working toward the same aim: saving lives.

## Relational conditions for sustained interprofessional collaboration

This category illustrates how interprofessional collaboration is strengthened over time through relational familiarity, co-location and structured opportunities for shared reflection and learning. These insights are reflected in the subcategories *Co-location promotes collaboration* and *Post-incident reflection and shared learning.*

### Co-location promotes collaboration

Familiarity among personnel was seen as a facilitator of trust at the scene, often enabled by recognising each other’s facial expressions. Being acquainted prior to an incident was considered advantageous and co-location, sharing the same physical space, was described as a means of fostering this familiarity.

*We are co-located with the ambulance service and I see that as a significant advantage. Recognising each other's facial expressions to some extent fosters an understanding between us, which in turn makes it easier to collaborate when we are out at the scene*. (F6)

Shared premises also allowed for spontaneous training opportunities, although participants noted that increased workloads have reduced the time available for such activities. Experience and confidence in the roles expected during major incidents were considered essential, especially given the infrequency of such events. Participants emphasised that these scenarios should be practised more regularly. Joint training sessions involving the police, fire brigade and ambulance service were described as rare, which participants viewed as a shortcoming. They highlighted the value of involving newly recruited police officers in such exercises to help them understand interprofessional collaboration and operational procedures during major incidents. Communication, both face-to-face and via radio, was described as an area of failure during training, attributed to a lack of joint practice. The fire brigade, which traditionally trains with a full command structure, also noted deficiencies in the command structures of the other two organisations.

### Post-incident reflection and shared learning

Post-incident evaluation, reflection and review were described as essential for developing understanding, answering questions and providing feedback. This process was considered beneficial for all parties involved, offering opportunities to learn from both personal and shared experiences across organisational boundaries.

*You address all questions: ‘Why did it take time?’ You figure out what could have been done better, while also focusing on what was done well*. (A3)

Although immediate evaluation following an incident was considered ideal, participants noted that routines differ between organisations, making this difficult to implement consistently. Unlike the ambulance service and police, the fire brigade was reported to have more established evaluation procedures, an advantage given their lower volume of emergency calls.

## Discussion

This study explores the experiences of collaboration among emergency first responders during major incidents. While the findings indicate that effective communication and a continuous flow of information are critical components of successful interprofessional collaboration, communication failure remains a significant challenge in emergency response settings [39].

Initial information provided by emergency dispatch centres and the first units to arrive at the scene is essential for preparing personnel prior to their arrival. However, mental preparedness can be compromised when this preliminary information is ambiguous. Moreover, such information may be inaccurate or misleading [24, 40], underscoring the importance of maintaining an open mindset to avoid fixation or misdirection during subsequent response efforts [9]. The findings further highlight the necessity of ongoing information dissemination throughout the operation to ensure that all participating organisations operate under consistent assumptions. In this context, the use of shared terminology is vital for fostering mutual understanding [41].

All three emergency response organisations in our study reported experiencing communication failures, often due to technical issues such as incorrect radio usage or equipment malfunction. Major incidents may require the use of multiple technological platforms, necessitating responders to engage with various devices to access relevant information and participate effectively in communication [41]. When radio communication is compromised, particularly due to user error, the transmission of information is severely impaired, limiting responders’ ability to engage in collaborative decision-making [42]. A lack of training in radio equipment operation was identified as a key contributor to communication breakdowns, a challenge documented in several major incidents, including the attacks in Utøya (Norway, 2011) and Brussels (Belgium, 2016), where communication failures were partly attributed to insufficient training [39]. Hence, recurrent training in communication technology for all emergency personnel is recommended. Simulation-based training has demonstrated improvements in radio communication performance and stress management, e.g., virtual training for fire brigade personnel improved communication strategies under stress. Future research could assess how such training reduces errors during real incidents [43].

Interorganisational communication failures can lead to delays in patient care and negatively impact patient safety [44], reinforcing the importance of early and effective interprofessional communication [41]. Ensuring continuous access to communication tools enhances organisational situational awareness. Conversely, when personnel are unable to communicate effectively, they risk exclusion from the information flow and collaborative processes. Therefore, both inter- and intra-professional communication should be regarded as pivotal to the management and outcomes of major incidents [39].

The findings also shed light on the impact of organisational differences in leadership and culture. Competent leadership is essential for facilitating effective interagency collaboration, as supported by Branderud et al. [45] and Andersson et al. [9]. Transparent leadership is particularly important in complex major incident scenarios, requiring clarity and decisiveness. Leadership effectiveness is enhanced by both experience and contextual familiarity and should be continuously developed [11]. In such contexts, leadership should focus less on operational minutiae and more on cultivating a strategic, high-level perspective that promotes a comprehensive understanding of the situation [11]. Differences in leadership perspectives may reflect whether the training is based on real incidents or simulation exercises*.* Leadership development programmes that incorporate simulation-based training have been shown to improve communication, stress management and strategic decision-making among emergency responders [46].

This study suggests that leadership is most effective when individuals have a clear understanding of their own roles and those of their colleagues, thereby strengthening interprofessional collaboration. A well-defined understanding of individual responsibilities improves conditions for managing major incidents [47]. Furthermore, achieving shared operational objectives depends on clear leadership that communicates mission goals and situational awareness to all involved parties. Shared objectives and teamwork are essential components of effective collaboration [9, 44, 47].

The findings also underscore the importance of personal familiarity among organisations, suggesting that such relationships are a key to successful incident management. Incident commanders reported that collaboration and leadership are more effective when personnel are personally acquainted [24]. Regular interaction among co-located emergency response personnel can strengthen interpersonal relationships and facilitate smoother collaboration [11], potentially improving incident management. However, high staff turnover [48–50] may hinder the maintenance of personal familiarity both within and across organisations. Structured networking opportunities, such as interprofessional educational activities or drills, have been highlighted as an important means of fostering professional relationships despite personnel changes [51].

Finally, the study reveals that collaboration and leadership are significantly challenged in scenarios characterised by safety concerns and unclear protocols. These challenges are particularly evident during incidents involving ongoing lethal violence, where stressful working conditions can lead to confusion among responders [39]. Security concerns in such environments pose heightened risks to healthcare personnel [39, 52]. In unsafe settings, operational priorities may shift rapidly, often focusing solely on life-saving interventions. These interventions may involve not only ambulance personnel but also support from the police and fire services [44], further necessitating effective collaboration.

Improved safety equipment and stronger collaboration with law enforcement are vital for ensuring the safety of ambulance personnel [3, 39]. When healthcare providers feel secure, they are better able to focus on their collaborative and leadership responsibilities, rather than being distracted by safety concerns or ethical dilemmas. As noted by Janairo et al. [53], major incidents introduce complex ethical considerations, with personnel often feeling a moral obligation to assist victims to the same extent as law enforcement, despite potential risks to their own safety. This perceived ethical duty can result in significant moral pressure. Establishing clear guidelines and protocols for navigating such situations may help alleviate this pressure and support more effective responses.

## Limitations

This study was guided by Lincoln and Guba’s [37] framework for trustworthiness, encompassing credibility, dependability, confirmability and transferability. Given the complexity of collaboration among emergency first responders during major incidents, methodological rigour was essential.

To ensure credibility, focus group interviews were selected in preference to individual interviews in order to capture shared experiences, challenge assumptions and explore interprofessional dynamics in situ. This format, inspired by Krueger’s [31] content analysis approach, encouraged interaction and reflection among participants from the ambulance service, fire brigade and police. Moderators with contextual familiarity but no hierarchical ties facilitated open dialogue, thereby reducing the risk of social desirability bias. Analytical credibility was further strengthened through peer debriefing and reflexive team discussions throughout the research process. Dependability was addressed by maintaining a clear audit trail of decisions made during the data collection and analysis. A consistent, iterative coding strategy guided by Krueger’s model was applied across all transcripts, with regular reassessment of categories to ensure coherence with the empirical material. To support confirmability, interpretations were grounded in the participants’ own words and the researchers’ preunderstandings were consciously bridled. Findings were substantiated with illustrative quotations and divergent views were reported to reflect the breadth of perspectives. Transferability, while inherently limited by context, was enhanced through rich descriptions of the study setting and participant composition. Purposeful sampling ensured diversity of roles and experiences, allowing insights that may be relevant to other multi-agency emergency settings. A possible limitation is that we only considered biological sex, without accounting for individuals who identify as non-binary or those who preferred not to disclose their gender [54]. Although participant numbers and representation varied across focus groups, potentially influencing group dynamics, the findings remained consistent between groups and the inclusion of all services in each group may have further strengthened transferability.

## Conclusions

This study highlights that effective collaboration among emergency first responders during major incidents should include coordinated communication, structured leadership and mutual understanding of each organisation’s professional roles. Communication failures, often described as stemming from technical limitations and insufficient radio system training, were identified as challenges that may adversely affect operational work and potentially compromise patient safety.

Structured leadership, characterised by clearly defined roles and responsibilities, was described as important for organising collaboration efforts and supporting operational work during major incidents. Safety concerns in incidents involving ongoing violence were highlighted as specific collaborative challenges that require proactive planning and clear protocols. The findings suggest that leadership development focusing on strategic decision-making and interagency coordination, supported by regular simulation-based exercises, may strengthen preparedness for such situations.

Personal familiarity and co-location among emergency service organisations were described as contributing to trust, collaboration and opportunities for shared learning and professional development. To maintain these benefits despite staff turnover, structured arenas for networking and interprofessional interactions may be valuable for interprofessional relationships.

Overall, the findings indicate that joint training initiatives and an interprofessional approach may play an important role in strengthening preparedness among first responders and supporting an effective emergency response during major incidents.

## Supplementary Information

Supplementary Material 1.

## Data Availability

The datasets generated and/or analysed during the present study are not publicly available due to participants’ confidentiality, but are available from the corresponding author on reasonable request.

## Abbreviations

EMS: Emergency Medical Services
METHANE: A mnemonic for: Major incident, Type of incident, Hazards, Access/egress, Number of casualties, Emergency services required
SBAR: Is a structured communication tool widely used in healthcare and the emergency services to ensure clear, concise and standardised information exchange.
RAKEL: Sweden’s national secure radio communication system for the emergency services and critical societal actors
