# Supplementary material for: Collaboration among emergency first responders at major incidents – an explorative focus group study
Source: Scand J Trauma Resusc Emerg Med. 2026 Apr 2;34:75. doi: 10.1186/s13049-026-01606-4 (PMC13081618; doi:10.1186/s13049-026-01606-4)
Supplement: Supplementary file 1 — Supplementary Material 1. [file 13049_2026_1606_MOESM1_ESM.docx]

**Participant Introduction**

- Please share your current role. (If you are a registered nurse, how many years have you worked in the profession? If you are a specialist nurse, how long have you held that role?)
- Your age?
- How many years have you been in operational service?
- Sex

**Opening Question**

When we talk about collaboration between ambulance services, police, and fire brigade during a major incident, what comes to mind for you?

**Core Discussion Topics**

- What experiences have you had in these situations?
- In your opinion, what are the most important factors for effective collaboration during a major incident?
- How would you describe situations where collaboration worked well, and those where it worked less well

**Examples of Prompts to Encourage Group Interaction**

- Could someone share an example of this?
- Does anyone else have a similar or different experience?
- What were your thoughts and feelings during that situation?
- In what context might this occur?
- Please elaborate—why do you think it happened that way?
- How do others in the group see this?

**Facilitator Tips**

- Encourage participants to build on each other’s comments.
- Ask for contrasting views to stimulate discussion.
- Use follow-up questions to deepen understanding.
